# Effects of Berberine against Pancreatitis and Pancreatic Cancer

**DOI:** 10.3390/molecules27238630

**Published:** 2022-12-06

**Authors:** Filip Vlavcheski, Eric J. O’Neill, Filip Gagacev, Evangelia Tsiani

**Affiliations:** 1 Department of Health Sciences, Brock University, St. Catharines, ON L2S 3A1, Canada; 2 Centre for Bone and Muscle Health, Brock University, St. Catharines, ON L2S 3A1, Canada

**Keywords:** pancreatitis, berberine, polyphenols, pancreatic cancer, in vitro, in vivo

## Abstract

The pancreas is a glandular organ with endocrine and exocrine functions necessary for the maintenance of blood glucose homeostasis and secretion of digestive enzymes. Pancreatitis is characterized by inflammation of the pancreas leading to temporary or permanent pancreatic dysfunction. Inflammation and fibrosis caused by chronic pancreatitis exacerbate malignant transformation and significantly increase the risk of developing pancreatic cancer, the world’s most aggressive cancer with a 5-year survival rate less than 10%. Berberine (BBR) is a naturally occurring plant-derived polyphenol present in a variety of herbal remedies used in traditional medicine to treat ulcers, infections, jaundice, and inflammation. The current review summarizes the existing in vitro and in vivo evidence on the effects of BBR against pancreatitis and pancreatic cancer with a focus on the signalling mechanisms underlying the effects of BBR.

## 1. Introduction

The pancreas is an ultrasensitive oblong-shaped gland located directly behind the stomach, or more specifically in the upper epigastrium and left hypochondriac regions of the abdomen. Due to its location, pancreatic diseases have been notoriously difficult to study and understand. Detailed examination and diagnosis of pancreatic ailments often involves highly specialized equipment and expertise which are not always readily available, especially in developing countries. Routine cost-effective imaging techniques such as transabdominal ultrasound are not very accurate in diagnosing pancreatic diseases as oftentimes the pancreas may be obscured by abdominal gas or other organs and cannot be completely visualized resulting in misdiagnosis/missed diagnosis [1]. Additionally, the symptoms of pancreatic diseases are often multifactorial, vague, and non-specific, resulting in poor diagnosis with only 9.7% of all pancreatic cancers being detected in the early stage [2]. More effective diagnostic techniques are expensive, invasive, and require access to pancreatic disease specialists with profound skills and knowledge which further complicates diagnosis. The pancreas is a chief organ performing endocrine functions such as insulin and glucagon (critical hormones involved in regulation of glucose homeostasis) secretion as well as exocrine functions such as secretion of digestive enzymes including amylase and lipase into the duodenum, thus aiding in the digestion of nearly 25,000 kg of consumed food throughout our lifetime [3]. With the involvement of revolutionary techniques in the field of genetics, molecular biology, and new in vitro and in vivo models of pancreatic diseases, the pancreas is now recognized as an organ that plays a life-sustaining role in the regulation and maintenance of normal physiological processes in various organ systems. These advances have enhanced our understanding of the physiology and pathophysiology of the pancreas, allowing for better understanding of previously enigmatic diseases and opening new avenues for disease treatment and prevention. Namely, recent developments in the field of biotechnology have allowed for the 3D bioprinting of pancreatic islets cells that successfully retained their morphology, function, and viability for up to 7 days in culture [4]. Increasing the number of transplanted islet cells would dramatically improve patient outcome and help achieve insulin independence, as often several islet cell infusions are required to reach significant clinical benefits [5]. Furthermore, significant progress has been achieved in bioprinting artificial pancreases made of biodegradable biopolymers with embedded bioactive factors and cells to promote growth and development [6]. However, despite 10 years of intensive research, bioprinted tissues have not reached adequate morphological and functional organicity to make them an effective substitute for allografts and further research is required [7].

### 1.1. Pancreatitis

Pancreatitis is a serious medical condition characterized by inflammation of the pancreas. The disease is classified into acute or chronic pancreatitis based on disease presentation and pathophysiology.

#### 1.1.1. Acute Pancreatitis

Acute pancreatitis (AP) is a rapid inflammatory disease of the pancreas with a variety of clinical and morphological presentations. AP patients present with onset of sudden severe epigastric pain that often radiates to the back, abdominal pain that gets worse after eating, abdominal tenderness, nausea, vomiting, fever, and rapid pulse [8]. Diagnosis is confirmed in accordance with the revised Atlanta criteria if at least two of the three criteria are satisfied: (1) abdominal pain; (2) serum lipase or amylase levels at least three times the upper physiological limit; and/or (3) radiographic evidence of AP performed by contrast-enhanced CT, or less often MRI or transabdominal ultrasound [8]. Symptoms are variable and clinical severity of AP is classified into three categories: mild, moderate, or severe. Mild AP is characterized by no local or systemic complications and severe AP is characterized by persistent single or multiple organ failure, usually with infected (peri)pancreatic necrosis [8].

AP is the number one cause for gastrointestinal-related hospital admissions in North America, posing a substantial burden to health care systems with annual incidence ranging from 13–45 cases per 100,000 people [9,10]. Additionally, AP is currently on the rise with an approximate 30% increase in the number of hospital admission in the last 10 years [11]. Despite the advancements in diagnostics, around 10–15% of AP cases remain unexplained or idiopathic [12,13]. Whether mild, moderate, or severe, AP usually requires hospitalization and close monitoring since complications may be unpredictable, sudden, and sometimes fatal [12]. Importantly, it should be emphasized that treatment options for AP remain extremely limited and many patients continue to experience multiple reoccurrences that prolong inflammation, fibrosis/scarring, and cause permanent damage to pancreatic tissues resulting in chronic pancreatitis (CP).

Although, all patients presenting with AP are hospitalized, there are currently no effective pharmacologic treatments for pancreatitis. Instead, treatment involves mostly supportive therapy including IV fluid resuscitation to prevent dehydration and lower inflammation, antiemetics, and pain medication, especially during the first attack in order to determine the specific cause [14]. The two most common aetiologies of AP are gallstones and excessive alcohol consumption [15]. Other subtypes of AP include hereditary, autoimmune, iatrogenic, cystic fibrosis, hyperlipidemia, and hypercalcemia-induced pancreatitis. AP may be treatable if the aetiology is known. For instance, autoimmune pancreatitis is usually treated with immunosuppressants (e.g. Prednisone, Azathioprine) or immunomodulating/biologic therapy (e.g. Rituximab) which often results in the pancreas returning to its normal/healthy state with no radiographic or histological changes [16,17]. However, the other subtypes may lead to serious conditions and even death, especially if the aetiology is unknown.

#### 1.1.2. Chronic Pancreatitis

Chronic Pancreatitis (CP) is a fibroinflammatory disease caused by repetitive episodes of pancreatic inflammation of variable intensity and duration leading to irreversible pancreatic damage and permanent loss of function [18]. The repetitive episodes of tissue inflammation lead to excessive fibrotic tissue buildup, exocrine and endocrine insufficiency, chronic pain, and significantly reduced quality of life and mental health [18]. Definitive CP may be diagnosed with imaging alone whereas diagnosis of suspected/probable CP requires the presence of clinical features (i.e., pain, nausea, vomiting, steatorrhea) in addition to imaging confirmation [18]. Definitive CP diagnosis involves radiographic evidence of parenchymal and intraductal pancreatic fibrosis, calcifications, endocrine and exocrine insufficiency resulting in diabetes, malnutrition, and steatorrhea. The symptoms of CP may have devastating effects on patients’ quality of life (QOL) and life expectancy is often inevitably reduced [18].

CP is very difficult to diagnose thus epidemiological studies are scarce with limited studies suggesting an annual prevalence of 123–143 cases per 100,000 people [19]. Early pancreatic changes are oftentimes not easily detectable with regular imaging techniques (e.g. MRI, CT) and even the most sophisticated tools currently available can fail to detect these early changes. Detailed investigations use endoscopic ultrasound techniques where a thin, flexible, ultrasound probe is inserted in the mouth and guided into the stomach to provide a better vantage point to observe the pancreas, allowing the pancreas to be more easily examined and biopsied using fine needle aspiration [20]. Alternatively, endoscopic retrograde cholangiopancreatography (ERCP) combines the use of an endoscope and X-ray, where a dye is injected directly into the ampulla of Vater allowing the pancreas to be visualized in detail. However, these techniques are costly, and require sedation and highly specialized personnel. Additionally, post-ERCP complications happen in 2–10% of patients and up to 40% of high-risk patients (e.g. young patients, history of post-ERCP complications, sphincter of Oddi disfunction) [21]. These complications often result in damage to the pancreas followed by a major pancreatitis episode, pancreatic necrosis, and organ failure. Therefore, understanding and detecting early pancreatic changes and using less invasive, readily available methods are imperative for the treatment, prevention, and improved understanding of the pathology and mechanism underlying CP.

#### 1.1.3. Pathophysiology of Pancreatitis

The first mention of the pathophysiology of pancreatitis dates back 120 years when Hans Chiari introduced the concept of pancreatic autodigestion [22]. Chiari postulated that pancreatitis is caused and driven by pancreatic juices, describing the pathomechanism of premature activation of pancreatic enzymes therefore contributing to disease severity and progression [22]. Under physiological conditions, enzymes are manufactured and released by pancreatic acinar cells as inactive proenzymes called zymogens. The zymogens are activated in the brush border of the duodenum where enteropeptidases cleave the NH_2_-terminal trypsinogen-activation peptide (TAP) from trypsinogen to form active trypsin that later catalyses the activation of the other zymogens. During the early stages of pancreatitis, zymogens are believed to be prematurely activated inside the pancreas causing inflammation, necrosis, and often severe damage to pancreatic tissue [23,24,25,26].

It is now established that several cellular events are central to the pathophysiology of pancreatitis including dysfunctional calcium signalling [27,28], mitochondrial dysfunction [29,30], premature trypsinogen activation within the acinar cells and macrophages [31,32,33], endoplasmic reticulum (ER) stress [34,35], impaired unfolded protein response (UPR) [36], and autophagy [37]. The aforementioned events may be triggered by common acinar cell toxins including alcohol, smoking (nicotine), and bile acids. Intraductal pressure caused by pancreatic duct obstruction, ductal cell exposure to bile acid, and luminal acidification may also exacerbate or trigger these events. Due to these changes/events, the immune system engages in crosstalk with acinar cells further intensifying inflammatory events.

#### 1.1.4. Cellular Mechanisms Leading to Acute Pancreatitis

**Figure 1:**
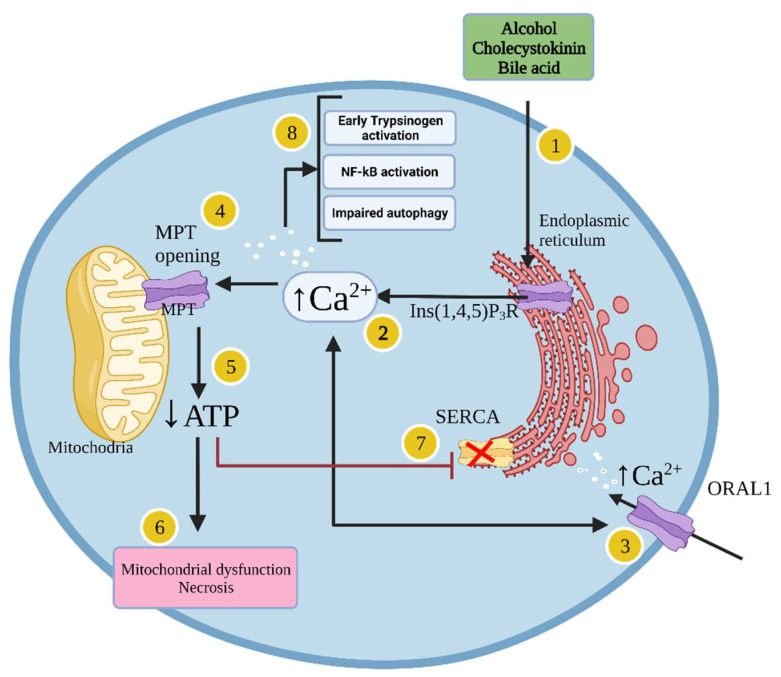
Mechanism of pancreatic acinar cell death induced by pancreatitis. In pancreatic cells, alcohol, cholecystokinin (CCK) and bile acids lead to activation of inositol 1,4,5-trisphosphate receptor (Ins (1,4,5) P3R) in the endoplasmic reticulum (ER) (1) which results in calcium release into the cytosol (2). The resulting low calcium concentration in the ER triggers opening of calcium release-activated calcium channel protein 1 (ORAL1), which allows extracellular calcium to enter the cell (3). This results in pathological global calcium concentration elevation within the pancreatic cells. Additionally, the calcium elevation results in the opening of mitochondrial permeability transition pores (MPTPs) (4) triggering a high conductive state and loss of mitochondrial membrane potential and depletion of ATP (5) leading to mitochondrial dysfunction and necrosis (6). The ATP reductions then inhibit the activity of the SERCA pump preventing the storage of excess cytosolic calcium ions in the ER (7). These events lead to early trypsinogen activation, NFκB activation, production of pro-inflammatory cytokines, impaired autophagy, and major dysfunction resulting in cell death (8).

In acinar cells, pancreatic insults such as exposure to alcohol or cholecystokinin (CCK) lead to inositol 1,4,5-triphosphate receptor-mediated calcium release from the endoplasmic reticulum (ER) (Figure 1) [38]. This results in low calcium concentration in the ER, triggering opening of calcium release-activated calcium channel protein 1 (ORAL1) to replenish the ER calcium concentration and allow calcium entrance from the extracellular space, leading to pathological global calcium concentration elevation [28]. This event results in opening of mitochondrial permeability transition pores (MPTPs) to a high conductive state, thus decreasing the mitochondrial membrane potential leading to mitochondrial dysfunction [27,39,40]. This event then leads to ATP depletion and impaired ATP-dependent mechanisms to reduce cytosolic calcium which further exacerbates calcium toxicity leading to necrosis (Figure 1) [41,42]. Pathological calcium elevations are dangerous and lead to activation of other cytotoxic pathways including premature trypsinogen activation, impairment in autophagy, and activation of inflammatory signalling including nuclear factor κB (NFκB) (Figure 1). Activation of the pro-inflammatory transcription factor NFκB in turn results in production of various mediators (e.g. cytokines) such as tumour necrosis factor-alpha (TNF-α), interleukin (IL)-1β, IL-6, and IL-18, among others, thus creating a potentially fatal inflammatory storm [43,44,45].

The location where the premature trypsinogen is activated and the fate of the activated trypsin during the early stages of pancreatitis are of vital importance and have garnered considerable interest from the scientific community. However, no clear understanding of these phenomena has been reached. Exploring these events in clinical pancreatitis has been extremely difficult since pancreatic tissue is not readily available for examination during early stages of pancreatitis in humans because obtaining pancreatic tissues is invasive and may produce additional complications for the patient. Due to these issues, understanding the details of pancreatitis has been limited. Additionally, due to lack of adequate models and the inability to keep isolated pancreatic acinar cells in culture for prolonged time, studies have mostly focused on AP [46]. However, there is a general understanding that these mechanisms are also involved in CP, although experimental evidence is clearly lacking.

### 1.2. Pancreatic Cancer

Pancreatic cancer, one of the deadliest and most aggressive forms of cancer, has a 5-year survival rate of 10%, and accounted for nearly half a million deaths globally in 2020 [47,48]. The prevalence of pancreatic cancer is currently on the rise and it is estimated to become the second to third most common cause of cancer-related death by the year 2030, mostly due to late diagnosis and limited treatment options [49]. Pancreatic cancer mainly affects the elderly with a median age at diagnosis of 71 and fewer than 20% of diagnoses occurring in those under the age of 60 [50]. Much of the mortality of PC is because few patients present with surgically respectable disease, patients rarely have symptoms before it develops into advanced stage disease, and any symptoms that may develop are usually non-specific and often overlooked [51].

#### 1.2.1. Pancreatic Cancer Risk Factors

The main modifiable risk factors for pancreatic cancer include obesity, type 2 diabetes, and tobacco use [51]. Development of pancreatic intraepithelial neoplasia (PanIN), the precursor to pancreatic ductal adenocarcinoma (PDAC), is largely correlated with fatty infiltration of the pancreas [52]. In a NIH cohort study, overweight or obese individuals had increased likelihood of developing pancreatic cancer with hazard ratios of 1.15–1.53 [53]. Patients with diabetes have double the risk of developing pancreatic cancer [54]. Additionally, new onset diabetes is an important risk factor and indicator of pancreatic cancer as about 1% of new onset diabetes diagnoses in adults over 50 are attributable to pancreatic cancer and patients with a new diabetes diagnosis (<1 year) have a 5.4-fold increase in relative risk of developing pancreatic cancer compared to a 1.5-fold increase in patients with long-standing diabetes [55]. Smokers are about two times as likely to develop pancreatic cancer compared with non-smokers, and pancreatic carcinomas from cigarette smokers harbour many more mutations than those from never-smokers; however, a specific genetic signature for smoking-related pancreatic cancer has yet to be identified [56]

An estimated 5–10% of pancreatic cancer cases can be attributed to genetic risk factors [57]. Peutz-Jeghers syndrome occurs due to a mutation in the tumour suppressor *LKB1* and individuals with Peutz-Jeghers syndrome have 35% increased risk of developing pancreatic cancer [58]. Hereditary breast–ovarian cancer syndrome, attributed to *BRCA1* or *BRCA2* mutations, is also associated with increased risk of developing pancreatic cancer. Mutation in *BRCA1* does not substantially increase the risk of developing pancreatic cancer, but *BRCA2* mutations are the most common genetic risk factor for pancreatic cancer with a relative risk of 3.5 [57,59]. Additionally, germline mutations in *CDKN2A* encoding the tumour suppressor p16 are associated with a 17% increased risk of developing pancreatic cancer [60]. Furthermore, CP is also a risk factor for pancreatic cancer and patients with hereditary pancreatitis associated with *SPINK1* and *SPINK2* mutations have a 40% lifetime risk of developing pancreatic cancer [58]. Chronic inflammation and fibrosis caused by CP can exacerbate the rate of malignant transformation significantly increasing the risk of developing pancreatic cancer [61]. A meta-analysis found that patients with CP have increased relative risk of pancreatic cancer (between 6.9 and 11.77) with the risk for pancreatic cancer in patients with hereditary pancreatitis increasing to 70 times in comparison to the control group [61,62].

#### 1.2.2. Molecular Characteristics of Pancreatic Cancer

**Figure 2:**
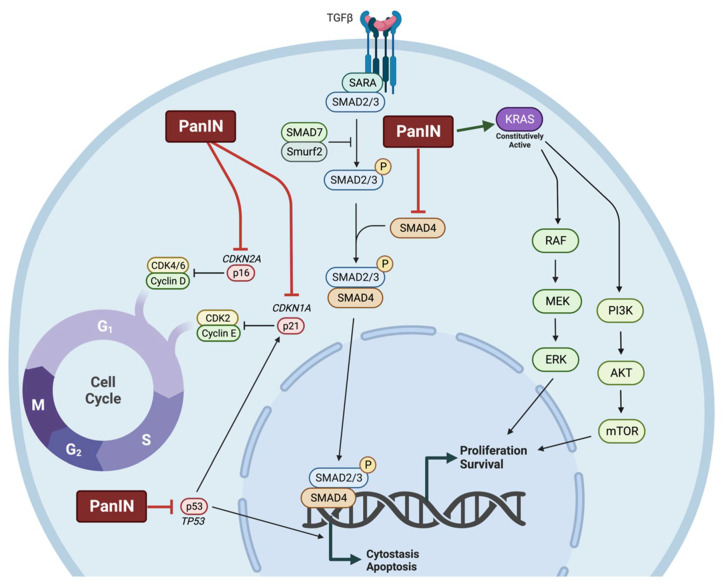
Cell signalling pathways disrupted in pancreatic intraepithelial neoplasia (PanIN). Pancreatic intraepithelial neoplasia is characterized by inactivating mutations in tumour suppressor *CDKN2A* and *CDKN1A*, encoding p16 and p21, leading to increased progression from G_1_ into S-phase of the cell cycle. Additionally, more than 90% of lesions have constitutively active KRAS mutations leading to overactivation of RAF-MEK-ERK and PI3K-AKT-mTOR signalling, resulting in enhanced proliferation and survival. Furthermore, PanIN interrupts downstream TGFβ signalling through SMAD4 inhibition leading to decreased apoptosis and cytostasis. Lastly, PanIN frequently results in loss-of-function mutations in the *TP53* gene encoding the tumour suppressor p53.

Most pancreatic cancers are ductal adenocarcinomas which arise from precancerous lesions known as pancreatic intraepithelial neoplasia (PanIN). These lesions are characterized by point mutations in the *KRAS* oncogene (present in ~90% of PDAC) responsible for constitutive activation of RAS and downstream PI3K-AKT-mTOR and RAF-MEK-ERK signalling, culminating in enhanced proliferation, survival, and motility [51]. Grade 1 PanIN is also characterized by telomere shortening which contributes to further mutation and cancer progression due to chromosomal instability [63]. Grade 2 lesions frequently have mutations leading to inactivation of the cyclin-dependent kinase inhibitors *CDKN2A* and *CDKN1A* encoding the proteins p16 and p21, respectively. Later stages of carcinogenesis present in grade 3 and grade 4 lesions include mutation to the tumour suppressor *TP53* and inactivating mutations in *SMAD4* contributing to unrestricted progression through the cell cycle (Figure 2) [64].

#### 1.2.3. Pancreatic Cancer Diagnosis and Treatment

The most common clinical features of pancreatic cancer at the time of diagnosis are abdominal pain, abnormal liver function, jaundice, new-onset diabetes, indigestion, nausea/vomiting, back pain, and weight loss [65]. Tumour location greatly influences the symptoms a patient may experience, with pancreatic head or neck tumours more likely to cause biliary obstruction resulting in jaundice while pancreatic body tumours often invade the local vasculature resulting in back pain [65]. The pancreatic tail has fewer anatomical neighbours and, as a result, pancreatic tail tumours typically grow unimpeded with symptoms only arising from sites of metastasis [51,65].

Diagnosis is typically achieved using a combination of symptomatology, imaging, and serum biomarkers. CT angiography using dual-phase pancreatic protocol is the recommended imaging technique and can detect pancreatic cancer with a sensitivity of 90% [66,67]. Alternatively, MRI and endoscopic ultrasound may also be used in some cases [68,69]. Carbohydrate antigen (CA) 19-9 is a validated serum biomarker for pancreatic cancer in symptomatic patients with a sensitivity of 79–81% and specificity of 82–90% [70].

The most commonly used method for staging pancreatic tumours is a four-tiered system based on resectability: resectable, borderline resectable, locally advanced, and metastatic [71]. For resectable and borderline resectable tumours, surgical resection is the only treatment with curative potential [72,73]. Pancreatic head tumours are usually resected with a pancreaticoduodenectomy (Whipple procedure) involving removal of the pancreatic head along with the duodenum, proximal jejunum, common bile duct, gall bladder, and part of the stomach [51]. Tumours of the pancreatic body or tail are treated with distal pancreatectomy combined with splenectomy [51]. Neoadjuvant or adjuvant chemotherapy alongside surgical resection is also commonly used. About one third of pancreatic cancer patients present with locally advanced disease and half of patients present with distant metastases. In these cases, systemic chemotherapy using gemcitabine, fluorouracil, nab-paclitaxel, FOLFIRINOX, or combinations thereof is the primary treatment option [51]. Despite these treatment options, the overall prognosis for pancreatic cancer patients is poor and more effective treatments are urgently needed.

## 2. Berberine

**Figure 3:**
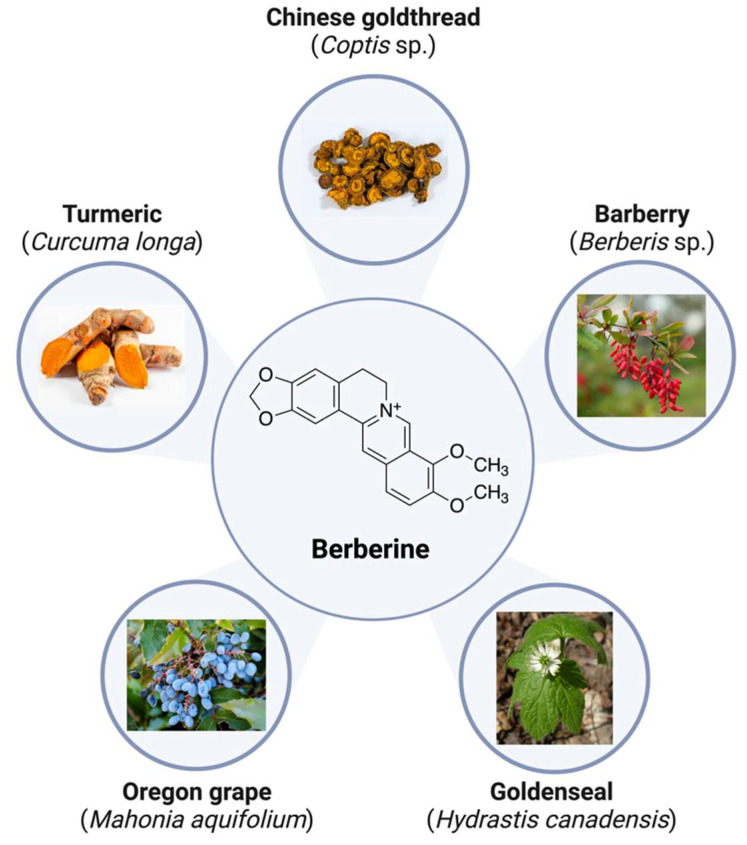
Chemical structure and sources of naturally occurring berberine (C_20_H_18_NO_4_^+^).

Berberine (BBR) is a naturally occurring plant-derived polyphenol that is present in a variety of plants/herbs including turmeric (*Curcuma longa*), barberry (*Berberis* sp.)*,* Chinese goldthread (*Coptis* sp.), goldenseal (*Hydrastis canadensis*), and Oregon grape (*Mahonia aquifolium*) [74]. Historically, extracts from these plants have been used in traditional Chinese medicine and by indigenous peoples of North America for a wide variety of ailments including ulcers, infections, jaundice, and inflammation [75]. In terms of chemical structure, BBR a is a pentacyclic isoquinoline alkaloid and quaternary ammonium salt (Figure 3) that has very poor solubility in water due to its several non-polar rings [76]. The poor solubility of BBR in water may be problematic and may prevent efficient absorption in the small intestine [77]. However, substantial progress has been achieved to significantly improve bioavailability [76,77].

BBR is found to exhibit potent anti-inflammatory [78,79], antioxidant [79,80], antidiabetic [81,82,83], and anticancer [84,85,86] properties in various tissues and organs. The current review summarizes recent findings on the effects of BBR on in vitro and in vivo models of AP, CP, and pancreatic cancer.

### Bioavailability of Berberine

**Figure 4:**
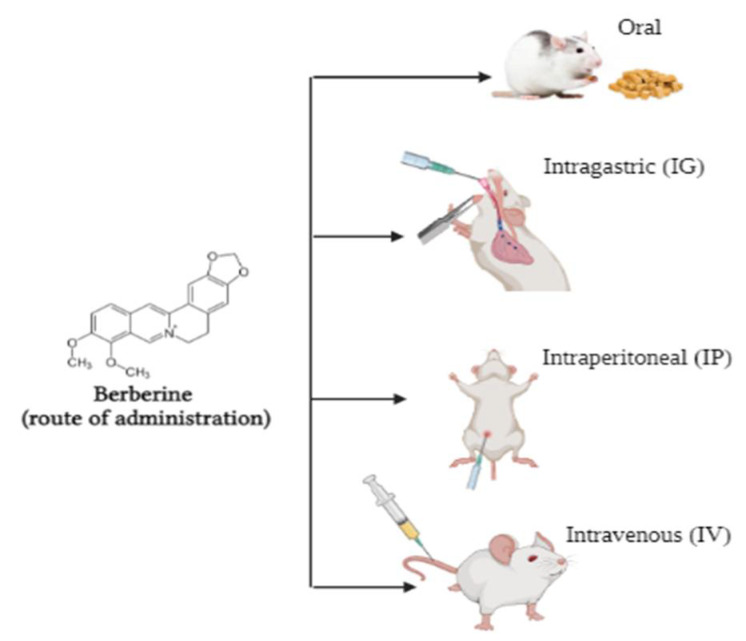
Different routes of Berberine administration. Common routes of BBR administration include oral, intragastric (IG), intraperitoneal (IP), and intravenous (IV).

Pharmacokinetic data obtained from rodents and humans have shown that BBR has poor absorption in the gut and a rapid metabolism in the body resulting in low bioavailability [87]. BBR is rapidly metabolized in the liver by oxidative demethylation and glucuronidation and its metabolites are excreted though the urine, bile, and faeces [88]. Under physiologic conditions, BBR is converted into an ionic form and self-aggregates resulting in reduced solubility and permeability in the gastrointestinal system [89]. Different routes of administration of BBR include oral (added in the diet or via an intragastric tube), intraperitoneal, or intravenous (Figure 4). A study showed that intragastric administration of 25 mg/kg BBR hydrochloride in male Sprague Dawley rats resulted in an area under the curve (AUC) of 2039.49 ng/mL/min with a C_max_ of 16.74 ng/m, indicating that BBR was not well-absorbed [90]. The total body clearance of BBR was 4999.51 L/h/kg, indicating that BBR is quickly removed from the body. Furthermore, BBR did not affect colonic motility, faecal pellet output, or colonic histology, suggesting that BBR is well-tolerated and causes no obvious side-effects in rats [90]. Another study found that intragastric administration of 20.8 g/kg or 41.6 g/kg single dose of BBR in mice resulted in blood plasma concentrations of 0.168 μg/mL (500 nM) and 0.432 μg/mL (1.2 μM), respectively, with toxicity observed at doses higher than 41.6 g/kg, indicating that oral administration of BBR is well-tolerated [91]. Similarly, intravenous administration of 9.0386 mg/kg resulted in blood plasma concentration of 0.4 μg/mL (1.2 μM) BBR and showed similar effects and toxicity as the intragastric route of administration. Additionally, BBR reached its peak blood concentration 1 h, 5 min, and 30 min after intragastric, intravenous, and intraperitoneal injection, respectively [91]. The data from the above studies indicate that when the concentration of BBR in plasma reaches a micromolar range, it is sufficient to elicit a response independent of the route of administration.

More importantly, in vivo distribution of BBR and its metabolites, thalifendine, berberrubine, and jatrorrhizine in various organs including heart, lungs, kidneys, brain, liver, fat, and muscle were found to be at least 10–30-fold higher than those in plasma 4 h after administration of 200 mg/kg oral dose of BBR dissolved in saline [92]. These results indicate that BBR and its metabolites might be exerting their effects by accumulating in the tissues rather than the plasma, which might explain the discrepancy between low plasma concentrations of BBR and substantial biological effects observed in vivo [92].

Finally, it should be noted, in a human pilot study, a single oral dose of BBR (400 mg) was given to 20 healthy volunteers resulting in mean maximum plasma concentration of only C_max_ 0.4 ng/mL and AUC(_0–1_) of 9.18 (ng/mL) × h [93].

In recent years, promising results have been achieved in developing techniques to increase bioavailability which may increase plasma concentrations of the drug by several hundred times. These techniques involve penetration enhancers or the use of lipid-and nano-particle delivery systems [94]. A study where BBR was immobilized onto a MgAl monolayer and administered to type 2 diabetic rats showed that the C_max_ and AUC levels were 4.23 and 4 times greater than the control, respectively [77]. Additionally, the total body clearance of MgAl-BBR was 55% lower than the control, indicating that MgAl-BBR may be present in the plasma for longer [77]. A similar study showed that poly lactic-co-glycolic acid (PLGA) nanoparticles prepared by encapsulating BBR with PLGA-doxorubicin conjugate (PDC) resulted in a 14-fold increase in the cytotoxicity in cancer cells in vitro (MDA-MB-231 and T47D) and in vivo in rats, and improved gut permeability by 5.5-fold and reduced P-gl efflux by 2-fold [95]. Elsheikh et al. showed that BBR-loaded chylomicrons enhanced ex vivo intestinal permeability by 10.5-fold and 2-fold in Caco-2 cells [96].

Additionally, most epithelial cells express P-glycoprotein (P-gp) on the plasma membrane which allows efflux of various drugs/foreign substances, including BBR, outside the cell resulting in limited oral bioavailability [97]. P-gp inhibitors are sometimes combined with BBR formulations to increase oral bioavailability [97]. These data indicate that various formulations could be utilized in designing BBR with enhanced bioavailability by using delivery systems (e.g. nanoparticles, chylomicrons) which would reach adequately high blood plasma and organ concentration of BBR and therefore optimize absorption.

## 3. Berberine and Pancreatitis

### 3.1. Effects of Berberine on Pancreatitis: In Vitro Studies

Exposure of palmitate treated pancreatic NIT-1 β cells to BBR (10 μM for 24 h) reduced triglyceride formation and fatty acid synthase (FAS) while significantly increasing AMPK expression, indicating that BBR could inhibit palmitate-induced lipid accumulation by decreasing fatty acid synthesis and increasing fatty acid oxidation by increasing the activity of the AMPK pathway [98].

A study has shown that exposure of MIN6 cells to amorphous solid dispersion of BBR (10 µM, 24 h) attenuated the palmitate-induced apoptosis, cytosolic cytochrome c and caspase 3, and mitochondrial potential, and increased cell viability and insulin secretion [99]. Furthermore, the effects of BBR were significantly reduced in iPLA2β-silenced MIN6 cells indicating that the cytoprotective effects of BBR may be partly attributed to the Ca^2+^-Independent Phospholipase A2 (iPLAβ) complex [99].

Furthermore, exposure of TGF-β1-stimulated M2 RAW 264.7 macrophages to 30 μM BBR for 24 h downregulated expression of CD206, a plasma membrane protein involved in the polarization of macrophages during cerulean-induced chronic pancreatitis [100].

In this review, we reported the current in vitro findings regarding the effects of BBR in pancreatic cells including the insulin-producing (endocrine) beta cells such as NIT-1and MIN6, pancreatic islets, and M2 RAW 264.7 monocyte/macrophages. Although these cells represent pancreatic endocrine cells and are not a true depiction of pancreatitis, the findings are relevant to pancreatitis. BBR is found to counteract cellular injury, the increased oxidative stress, and inflammation in beta cells [101] suggesting restoration of normal function. Furthermore, it is relevant to mention that the pancreatic acinar cells are the functional unit of the exocrine pancreas and are considered the initiating site of injury in pancreatitis [102]. However, in vitro models of pancreatitis lack the full inflammatory or systemic components of the disease which renders their use relatively limited, therefore there are currently no studies describing the effects of BBR in acinar cells and models of pancreatitis [102].

Although the solubility of BBR is poor and the compound is classified as “slightly soluble in water” the technical data indicate solubility of 1 mg/mL which is equivalent to 2.973 mM of BBR. In the studies summarized in the current review, the concentrations of BBR used to treat cells in culture range from 1 μM to 200 μM. These concentrations are much below the 2.973 mM value/solubility in water and indicate that BBR could be successfully dissolved in aqueous solutions/cell culture media. In addition, a study found that the solubility of BBR in aqueous solution is increased by 62% when the temperature is raised from 25 to 37 °C [103]. All in vitro experiments are performed at 37 °C and therefore the solubility of BBR is expected to not be an issue.

### 3.2. Effects of Berberine against Pancreatitis: In Vivo Studies

Sprague Dawley rats were intragastrically administered BBR (50 mg/kg) daily for 5 days followed by 3% sodium taurocholate injection (1 mL/kg) to induce severe acute pancreatitis (SAP) [104]. BBR was unable to attenuate histologic changes to the pancreas but ameliorated other SAP symptoms such as intestinal mucosal barrier damage. Rats with SAP had increased serum DAO activity, increased serum endotoxin levels, and increased bacterial translocation—markers of intestinal barrier dysfunction—however, pre-treatment with BBR significantly reduced these effects [104]. Furthermore, pathological scoring of the pancreas indicated pre-treatment with BBR exhibited a protective effect against injury to the ileal mucosa due to SAP. Lastly, BBR treatment was found to inhibit SAP-induced myosin light chain phosphorylation, which is an indicator of intestinal barrier dysfunction [104] (Table 1).

Pre-treatment of C57BL/6 mice with BBR (1–10 mg/kg) for 1 h reduced pancreatic edema and inflammation caused by cerulein (a CKK analogue)-induced AP (50 µg/kg/h for 6 h) [105]. Additionally, BBR pre-treatment exhibited a protective effect against lung damage caused by edema and inflammatory cell infiltrate. Interestingly, lower doses (1 mg/kg) of BBR exhibited no protective effect while higher doses (20 mg/kg) had less protective effects due to toxicity [105]. BBR-treated mice also had lower serum lipase and amylase activity as well as reduced myeloperoxidase (MPO) activities in the lungs and pancreas compared to untreated mice. Pre-treatment with BBR also reduced the production of the inflammatory mediators iNOS and NO, and reduced mRNA expression of TNF-α, IL-1β, and IL-6 [105]. The same results were also observed in L-arginine-induced SAP, confirming that BBR has general protective effects against AP. BBR pre-treatment inhibited the activation of JNK, a MAPK involved in the induction of inflammatory mediators, in both the cerulein and L-arginine-induced models of AP [105]. Treatment with a JNK inhibitor had the same protective effects against cerulein-induced AP as BBR, suggesting that the protective effects of BBR are linked to downregulation of the JNK pathway. Cerulein-induced pancreas and lung injury were also attenuated if BBR was administered 1 or 3 h following cerulein injection [105] (Table 1).

Intraperitoneal administration of BBR (10 mg/kg/b.w) in choline-deficient ethionine-supplemented (CDE) diet–induced SAP for 3 days inhibited histological damage to the pancreas and lung, and significantly reduced serum amylase and lipase levels, neutrophil sequestration in the pancreas assessed by myeloperoxidase activity, pancreatic mRNA cytokine expression (TNF-α, IL-1β, and IL-6), and mortality rate by 60% [106]. Furthermore, administration of BBR inhibited activation of nuclear factor kappa B, c-Jun N-terminal kinases, and p38 in the pancreas [106] (Table 1).

Intragastric administration of BBR (100 mg/kg) to Wistar rats daily for six days following SAP induction with L-arginine (3 g/kg) improved survival and protected the rats from pancreatic encephalopathy, a life threatening complication that arises from SAP [107]. BBR treatment reduced histopathological signs of inflammation and necrosis in the pancreas due to L-arginine-induced SAP and lowered serum amylase levels. In a context fear conditioning test, rats with SAP had decreased freezing time indicating hippocampus-dependent long-term memory deficits; this deficit was significantly attenuated by BBR treatment. BBR treatment also protected against SAP-induced increase in blood brain barrier permeability as assessed using Evans blue dye in circulation [107]. Additionally, SAP caused increased levels of proinflammatory cytokines (TNF-α and IL-1β) in brain tissue, however, BBR treatment restored these levels to baseline. Hippocampal tissues of SAP rats had increased levels of the apoptosis effector caspase-3 and increased levels of the neuronal necroptosis inducers RIP1 and RIP3 but BBR protected against SAP-induced increases in these proteins in the hippocampus [107] (Table 1).

Daily intraperitoneal injection with BBR (10 mg/kg) for 21 days in male Swiss albino mice attenuated cerulein-induced CP and fibrosis [100]. CP was induced by six intraperitoneal injections of cerulein (50 μg/kg/h) three days a week for 21 days. Treatment with cerulein reduced pancreatic weight and increased in plasma lipase and amylase levels, however, BBR attenuated these effects. Additionally, BBR treatment reduced cerulein-induced oxidative-nitrosative stress as indicated by reduced levels of pancreatic MDA and nitrate with increased levels of GSH [100]. Cerulein-treated mice had increased levels of pro-inflammatory (TNF-α, IL-6 and IL-1β) and profibrotic (TGF-β1) cytokines but BBR dose-dependently attenuated these effects. Treatment with BBR also attenuated cerulein-induced histopathological changes to the pancreas which included increased collagen deposition, inflammatory cell infiltration, acinar cell atrophy, and exocrine pancreas vacuolization [100]. Additionally, BBR dose-dependently reversed cerulein-induced increases in pancreatic fibrosis markers (α-SMA, collagen1a, collagen3a, and fibronectin) as indicated by western blotting and immunohistochemistry. Treatment with BBR upregulated AMPK signalling and downregulated TGF-β1/SMAD signalling [100]. BBR also increased expression of protective Smad7, upregulated E-cadherin (reduced EMT), and inhibited the transcription factors Slug and Snail. Cerulein treatment caused increased CD206 expression indicating M2 macrophage polarization, however, BBR significantly attenuated this effect [100] (Table 1).

## 4. Berberine and Pancreatic Cancer

### 4.1. Effects of Berberine against Pancreatic Cancer: In Vitro Studies

Treatment of BxPC-3 human pancreatic adenocarcinoma cells and HPDE-E6E7c7 normal human pancreatic ductal epithelial cells with BBR (10–200 µM) for 24–72 h showed that high concentration of BBR can inhibit pancreatic cancer cell growth and trigger caspase-independent cell death [108]. BBR (10–200 µM) treatment for 24–72 h caused a concentration and time-dependent reduction in cell proliferation and BxPC-3 cells were more sensitive to the cytotoxic effect of BBR after 24 h than HPDE-E6E7c7 cells. BBR (150–200 µM) treatment significantly increased caspase-3- and-7 activity in both cell lines (although activation was much greater in HPDE-E6E7c7 cells) and caspase inhibition using Z-VAD-FMK rendered cells less susceptible to BBR, suggesting that BBR induces apoptosis of cancer cells at high concentrations [108]. Immunostaining showed that treatment with 200 µM BBR for 24 h caused translocation of apoptosis-inducing factor (AIF), a caspase-independent death effector, from the mitochondria to the nucleus indicating that BBR also induces caspase-independent mechanisms of apoptosis. Visualisation of live cells treated with fluorescent BBR for 24 h showed that at low concentrations (10–50 µM) BBR is localised to the mitochondria, but at higher concentrations (100–200 µM) BBR localisation extends into the cytoplasmic and nuclear compartments [108] (Table 2).

Lovastatin is a 3-hydroxy-3-methylglutaryl-coenzyme A (HMG-CoA) reductase inhibitor that blocks cholesterol synthesis and is predominately used to treat cardiovascular disease. Panc02 cells were treated with increasing concentrations of BBR (0–5 µM) or lovastatin (0–0.12 µM) for 48 h, alone and in combination, and cell viability was assessed with crystal violet staining [109]. Both drugs showed dose-dependent inhibition of cell viability and the combination of both drugs had highly synergistic cytostatic/cytotoxic effects. BBR or lovastatin treatment alone did not affect cell cycle distribution, but combined treatment resulted in a two-fold increase in the percentage of cells in sub-G_1_ phase and a modest increase in the percentage of cells in G_1_ phase. Interestingly, pre-treatment with products of the mevalonic acid pathway (i.e., restoring cholesterol synthesis) attenuated the anticancer effects of lovastatin but not BBR, indicating that BBR enhances the anticancer effects of lovastatin independent of the cholesterol synthesis pathway [109] (Table 2). Additionally, inhibiting farnesyltransferase or geranylgeranyltransferase did not potentiate the effects of BBR further, suggesting that the effects of BBR against pancreatic cancer do not rely on inhibition of protein prenylation. Overall, this study indicates that BBR can slow the growth of pancreatic cancer cells in vitro through a mechanism that does not involve the cholesterol synthesis pathway.

BBR treatment of PDAC cells (PANC-1 and MiaPaCa-2) for 17–72 h inhibited proliferation and DNA synthesis, and increased the population of cells in the G_1_-phase of the cell cycle with a concomitant reduction in the S and G_2_/M-phase population [110]. Furthermore, BBR caused a decrease in mitochondrial membrane potential and a concentration-dependent decrease in ATP levels to a similar degree as metformin, an established mitochondrial complex I inhibitor with tumour-suppressive effects [110,111].These changes in mitochondrial function coincided with a concentration-dependent increase in phosphorylated levels of ACC (Ser^79^) and AMPK (Thr^172^), suggesting that BBR supresses the growth of PDAC cells through a mechanism involving decreased mitochondrial function leading to decreased ATP levels and activation of AMPK. BBR blocked neurotensin- and insulin-induced ERK and mTORC1 activation in a concentration-dependent manner as indicated by decreased phosphorylation of ERK (Thr^202^/Tyr^204^), p70S6k (Thr^389^), and S6 (Ser^240/244^). Interestingly, siRNA knockdown of AMPK blocked the effects of low dose BBR (<3µM) and only partially blocked the effects of higher concentrations (3–6 µM). Overall, these data provide evidence that BBR impairs mitochondrial function of PDAC cells and the effects of BBR against PDAC rely on AMPK-dependent and independent mechanisms (Table 2).

Cancer stem cells (CSCs) play a major role in the initiation, growth, and metastasis of tumours since they have similar characteristics to ordinary stem cells including multipotentiality and high capacity for self-renewal [112]. One method for identifying CSCs is to identify side population (SP) cells based on their ability to exclude Hoeschst dye, a characteristic associated with stemness [112]. Treatment of PANC-1 cells with BBR decreased the proportion of SP cells from 9.7 to 5.7% and this corresponded with down-regulation of stem cell-associated genes: SOX2, OCT4, and NANOG [113]. MIA-PaCa-2 cells had no SP cells in the control group but still had down-regulation of stem cell-associated genes following treatment with BBR [113] (Table 2). Overall, these results indicate that BBR may reduce initiation, growth, and metastasis of tumours by reducing stemness of pancreatic cancer cells.

Treatment of PANC-1 and Mia-PaCa-2 pancreatic cancer cells with 1–15 µM BBR for 72 h resulted in concentration-dependent inhibition of cell growth with IC_50_ values of 15 µM and 10 µM, respectively [114]. BBR significantly increased the G_1_ phase population of PANC-1 cells with a concomitant reduction in S phase population. Additionally, BBR treatment induced apoptosis of PANC-1 and Mia-PaCa-2 cells as indicated by increased Annexin V/PI staining and increased caspase-3/7 activity with 24 h and 48 h treatment but not 72 h treatment [114]. The proapoptotic effect of BBR coincided with a concentration-dependent increase in intracellular ROS levels suggesting that the effects of BBR may be ROS-dependent [114] (Table 2).

BBR was found to localize to the cytoplasm of MiaPaCa-2 cells following 1 h treatment with 10 µM concentration, and at 50 µM or 150 µM concentrations BBR is also found in the nucleus [115]. Furthermore, the localization of BBR is maintained after 48 h of treatment, and 48 h treatment with BBR (0.4–50 µM) caused a concentration-dependent reduction in cell viability. Use of the mitochondrial tracer tetramethyl rhodamine methyl ester (TMRM) showed that BBR localizes to the mitochondria and 50 µM BBR (48 h) was found to decrease citrate synthase activity, suggesting that BBR impairs mitochondrial function [115]. Additionally, 10 µM BBR (48 h) was sufficient to induce G_1_ cell cycle arrest and significantly decrease the S phase population. Furthermore, 48 h treatment with 10 µM and 50 µM caused a 20- and 33-fold increase in mRNA expression of cyclin dependent kinase inhibitor 1A (P21)—a marker of cellular senescence—and this coincided with a concomitant increase in senescence-associated β-galactosidase staining and increase in apoptotic caspase-3 activity [115]. BBR treatment was also found to induce autophagy of Mia-PaCa-2 cells as indicated by increased mRNA expression of LC3 and Beclin-1, and increased protein expression of LC3-I and LC3-II [115]. BBR treatment of Mia-PaCa-2 cells also decreased cell migration in a wound-healing assay and decreased invasion in a transwell assay which coincided with decreased mRNA expression of C-X-C motif chemokine receptor 4 (CXCR4) involved in migration and increased mRNA expression of death-associated protein 1 (DAP1) [115]. DNA methyltransferases (DNMT) epigenetically regulate enzymes involved in DNA repair mechanisms [116]. BBR treatment upregulated mRNA expression of DNMT1, DNMT3A, DNMT3B, and O6-methylguanine DNMT (MGMT) [115] (Table 2).

Treatment of Panc-1 pancreatic cancer cells with BBR (1–30 µM) for 72 h caused a concentration-dependent decrease in cell viability with an IC_50_ of 4.76 µM and treatment with 10 µM BBR for 48 h caused significant inhibition of cell metastasis in a transwell assay [117]. BBR treatment significantly decreased expression of tumour necrosis factor α (TNFα), carbohydrate antigen 242 (CA242, a diagnostic and poor prognostic biomarker of pancreatic cancer [118]), and the oncogenic protein K-Ras. Conversely, BBR upregulated the expression of the tumour suppressor cyclin dependent kinase inhibitor 2A (CDKN2A). Metabolomic analysis revealed that BBR dysregulates pancreatic cancer cell metabolism with a similar regulatory pattern to the pancreatic cancer drug gemcitabine, but the effect of BBR on cell metabolism was much stronger than that of gemcitabine. Additionally, BBR increased energetic metabolism (i.e., glycolysis and glutamine)-associated metabolites and decreased citric acid cycle-associated metabolites. Transmission electron microscopy confirmed that these metabolic changes were due to BBR-induced mitochondrial damage and this damage coincided with decreased citrate metabolism [117] (Table 2). Overall, these data indicate that BBR inhibits pancreatic cancer cell viability through a mechanism that likely involves mitochondrial damage leading to decreased citrate metabolism and disruption of fatty acid biosynthesis, which has an important role in the proliferation and metastasis of pancreatic cancer cells.

BBR treatment (1–2000 nM, 96 h–2 weeks) inhibited colony formation of MIA-PaCa-2 pancreatic cancer cells and inhibited the viability of MIA-PaCa-2, PANC-28, and AsPC-1 in a concentration-dependent manner with IC_50_ values of 1700 nM, 2000 nM, and 2000 nM, respectively [119]. MIA-PaCa-2 cells possess a R248W *TP53* gain-of-function mutation resulting in expression of a p53 protein with a mutated DNA binding domain effectively blocking the tumour suppressive properties of p53 [120]; transfecting MIA-PaCa-2 cells to express wildtype p53 caused a three-fold reduction in the IC_50_ of BBR [121]. Furthermore, BBR sensitized MIA-PaCa-2 cells to the MDM2 inhibitor Nutlin-3a [122]. MIA-PaCa-2 cells transfected with kinase-dead GSK-3β had more than three-fold greater sensitivity to BBR-induced inhibition of cell viability compared to cells expressing wildtype GSK-3β [123]. Similarly, BBR decreased colony formation to a much greater extent in kinase-dead GSK-3β mutants compared to cells expressing wildtype GSK-3β [123]. Overall, these studies provide evidence of the effectiveness of BBR against pancreatic cancer in vitro and suggest that expression of wildtype p53 and/or inhibition of MDM2 renders pancreatic cancer cells more sensitive to BBR, and that GSK-3β may be an important regulator of the sensitivity of pancreatic cancer cells to BBR (Table 2).

In another study, BBR (0–30 µM; 24 h) had no effect on proliferation and migration of pancreatic cancer cells (AsPC-1 and SW1990); however, BBR (3–30 µM; 20 h) did inhibit trans-endothelial migration of AsPC-1 cells, indicating that BBR may exert its anticancer effects by improving the lung vascular barrier [124]. TGF-β1 is involved in disruption of the endothelial barrier and its receptor (TGFBR1) is a predicted target of BBR based on motif-based screening in pharmacophore databases [125,126]. Pancreatic cancer cells (PANC-1, SW1990, and AsPC-1) expressed higher levels of TGF-β1 compared to normal pancreatic ductal epithelial cells, and inhibition of TGFBR1 in endothelial cells blocked the inhibitory effect of BBR on trans-endothelial migration of AsPC-1 cells [124]. Additionally, BBR caused a concentration-dependent decrease in levels of phosphorylated smad3, phosphorylated smad2, SNAIL1, and SLUG in endothelial cells, which are all downstream targets of TGF-β1 signalling. Using surface plasmon resonance and molecular docking studies, it was determined that BBR binds to TGFBR1 with an equilibrium dissociation constant of 18.0 µM, BBR inhibits TGFBR1 kinase activity in a concentration-dependent manner with an IC_50_ of 7.056 µM, and that BBR docks into the active site of TGFBR1 interacting with key residues including Glu45, Tyr49, Asp81, Tyr82, and His83 [124]. Overall, these data indicate that BBR reduces metastasis of pancreatic cancer cells by interacting with the intracellular kinase domain of TGFBR1 to prevent TGF-β1-induced damage to endothelial barrier (Table 2).

Primary acinar cells were treated with TGF-β (5 ng/mL; 2 days) to induce acinar-to-ductal metaplasia (ADM) and concurrently (2 days) or subsequently (1 day) treated with 10 µM BBR [127]. BBR attenuated induction of ADM in TGF-β-treated cells, increased levels of amylase, and decreased CK19 levels closer to basal [127]. In the early stages of PanIN, but before development of PDAC, there is a metabolic shift towards glycolysis known as the Warburg effect [128]. TGF-β-treated acinar cells had increased glucose consumption and lactate production, markers of glycolysis, but BBR restored these levels to basal and the glycolysis-supressing effect of BBR was comparable to 2-deoxy-D-glucose (2-DG), a glycolysis inhibitor [127]. Additionally, both BBR and 2-DG decreased glycolysis-related gene expression: lactate dehydrogenase (LDHA), aldolase A (ALDOA), live phosphofructokinase (PFKL), pyruvate kinase M2 (PKM2), and 3′-phosphoinositide-dependent kinase 1 (PDK1). Interestingly, BBR increased activated levels of AMPK, and decreased levels of active mTOR and HIF-1α [127]. Compound c, an AMPK inhibitor, restored glycolysis and prevented BBR from suppressing the development of PanIN [127]. Taken together, these results indicate that BBR can prevent and, to some extent, reverse PanIN due to CP by activating AMPK and supressing the Warburg effect. Furthermore, treatment of MIN6 insulinoma cells with BBR (2.5–50 µM, 2–24 h) resulted in concentration-dependent reduction in cell viability with an IC_50_ of 5.7 µM for 16 h treatment and this coincided with a concentration-dependent increase in the population of apoptotic cells as indicated by increased Annexin V staining and increased DNA fragmentation [129]. These effects of BBR coincided with increased levels of pro-apoptotic proteins including increased cytoplasmic cytochrome C, AIF, Apaf-1, Bax, cleaved caspase-3, and cleaved PARP, and decreased expression of the antiapoptotic marker Bcl-2 [129] (Table 2). Ultimately, these data show that BBR stimulates apoptosis of insulinoma cells.

**Table 2:** Effects of BBR against pancreatic cancer in vitro.

Model	Treatment	Effects	Signalling	Ref.
BxPC-3	10–200 µM BBR 24–72 h	↓ Proliferation	↑ Caspase 3/7	[108]
Panc02	0–10 µM BBR 0–0.12 µM Lovastatin 48 h	↓ Cell viability	-	[109]
		↑ Sub-G_1_ and G_1_ phase populations		
PANC-1 MiaPaCa-2	0.3–6 µM BBR 17–72 h	↓ DNA synthesis	↑ pAMPK (Thr^172^) ↑ pACC (Ser^79^) ↓ mTORC1 ↓pp70 S6K (Thr^389^) ↓ pS6 (Ser^240/244^) ↓ pERK (Thr^202^/Tyr^204^) ↑ pRaptor (Ser^792^)	[110]
		↓ Proliferation		
		↑ G_1_-phase population		
		↓ S and G_2_/M-phase population		
		↓ Mitochondrial membrane potential		
		↓ ATP levels		
Mia-PaCa-2 PANC-1	15 µM BBR 72 h	↓ CSC population	↓ SOX2 ↓ OCT4 ↓ NANOG	[113]
PANC-1 Mia-PaCa-2	1–15 µM BBR 72 h	↓ Cell viability	↑ Caspase-3/7 activity	[114]
		↑ G_1_-phase population		
		↓ S-phase population		
		↑ Apoptosis		
		↑ ROS		
Mia-PaCa-2	10–50 µM BBR 1–48 h	↓ Cell viability	↑ p21 ↑ Caspase-3 activity ↑ LC3 ↑ DAP1 ↓ CXCR4 ↑DNMT1 ↑DNMT3A ↑ DNMT3B ↑ MGMT	[115]
		BBR mitochondrial localisation		
		↓ Citrate synthase activity		
		↑ G_1_-phase population		
		↓ S and G_2_-phase population		
		↑ Senescence		
		↓ Migration		
		↓ Invasion		
Panc-1	1–60 µM BBR 48–72 h	↓ Cell viability	↓ TNFα ↓ CA242 ↓ K-Ras ↑ CDKN2A	[117]
		↑ Apoptosis		
		↓ Metastasis		
		↑ Glycolysis-associated metabolites		
		↑ Glutamine-associated metabolites		
		↓ Citric acid cycle-associated metabolites		
		↑ Mitochondrial damage		
		↓ Citrate metabolism		
MIA-PaCa-2 PANC-28 AsPC-1	1–2000 nM BBR 72 h–2 weeks	↓ Cell viability	-	[119,121,122,123]
		↓ Colony formation		
PANC-1 AsPC-1 SW1990	0–30 µM BBR 24 h	↓ Trans-endothelial migration	↓ pSmad2 ↓ pSmad3 ↓ SNAIL1 ↓ SLUG	[124]
TGF-β-treated Primary acinar cells	ADM induction: 5 ng/mL TGF-β 2 Days 10 µM BBR 1–2 days	↓ PanIN	↓ CK19 ↓ LDHA ↓ALDOA ↓ PFKL ↓ PKM2 ↓ PDK1 ↑ pAMPK ↓ pmTOR ↓ HIF-1α	[127]
		↓ ADM		
		↓ Glycolysis		
MIN6	2.5–50 µM BBR 2–24 h	↓ Cell viability	↑ Cytochrome C ↑ AIF ↑ Apaf-1 ↑ Bax ↑ Cleaved Caspsase-3 ↑ Cleaved PARP ↓ Bcl-2	[129]
		↑ Apoptosis		
		↑ DNA fragmentation		

Table legend: ↑ increases, ↓ reduces.

### 4.2. Effects of Berberine against Pancreatic Cancer: In Vivo Studies

Treatment of Panc02 xenografted C57/B16 mice with BBR (oral administration, 100mg/kg/day) or lovastatin (intraperitoneal injection, 30 mg/kg/day) for 14 days slightly decreased tumour volume when given alone, but significantly decreased tumour volume when given in combination [109] (Table 3). Ultimately, this study shows that BBR administered orally reaches high enough concentrations in vivo to slow tumour growth and that BBR acts synergistically with cholesterol-lowering medications such as lovastatin.

Intraperitoneal injection of BBR (5 mg/kg/day) decreased the weight and volume of MiaPaCa-2 tumour xenografts in nude mice by approximately 70% [110]. Additionally, this dose of BBR was well-tolerated by the mice with no noticeable side-effects and no change in body weight. Interestingly, the tumour suppressive effects of BBR were similar to the antitumour effects of metformin at 250 mg/kg/day [110] (Table 3).

Mice that underwent intragastric administration of BBR (100–200 mg/kg/day) had a dose-dependent increase in overall survival after being intravenously injected with AsPC-1 cells through the tail vein [124]. Furthermore, the mice administered BBR (200 mg/kg/day) had 36.5% fewer lung metastases and 35% lower overall area of metastatic nodes in the lungs after six weeks. Ki-67 staining of lung metastases showed no changes between BBR-treated and control mice, indicating that BBR did not significantly affect the proliferation of tumour cells in lung metastases. In order to assess lung infiltration of circulating tumour cells, researchers labelled AsPC-1 cells with carboxyfluorescein succinimidyl ester (CFSE) prior to injecting them into the tail vein of mice; 24 h later, the lungs were homogenized to create a single cell suspension that was analysed using flow cytometry which revealed that BBR reduced accumulation of pancreatic cancer cells in the lungs, and 3D reconstruction fluorescent images of lung tissue sections showed that the cancer cells that did accumulate in the lungs were outside of the microvessels, suggesting that BBR may block transportation of tumour cells from circulation into the interstitial fluid [124]. As mentioned in the previous section, TGFBR1 was found to play a crucial role in the antimetastatic effect of BBR in vitro, and inhibition of TGFBR1 activation in vivo using A83-01 (10 mg/kg/day) also suppressed the antimetastatic effect of BBR [124] (Table 3).

Induction of CP in C57BL/6 mice using cerulein (50 µM/kg, 6 h/day, 3 days/week, 8 weeks) resulted in acinar-to-ductal metaplasia (ADM) after 4 weeks and pancreatic PanIN after 8 weeks [127]. BBR treatment (200 mg/kg/day, 3 days/week) for 4 weeks following induction of CP decreased the area of preneoplastic lesions. BBR given for 8 weeks alongside cerulein had a greater therapeutic effect than BBR administered after establishment of CP as indicated by inhibition of the development of ADM, PanIN, and fibrosis [127]. Mice with CP had decreased levels of serum amylase which is characteristic of damaged acinar cells in PanIN lesions [130], however, BBR treated mice had amylase levels closer to basal [127] (Table 3). Overall, these results show that BBR can prevent and partially reverse neoplastic lesions in the pancreas caused by CP.

**Figure 5:**
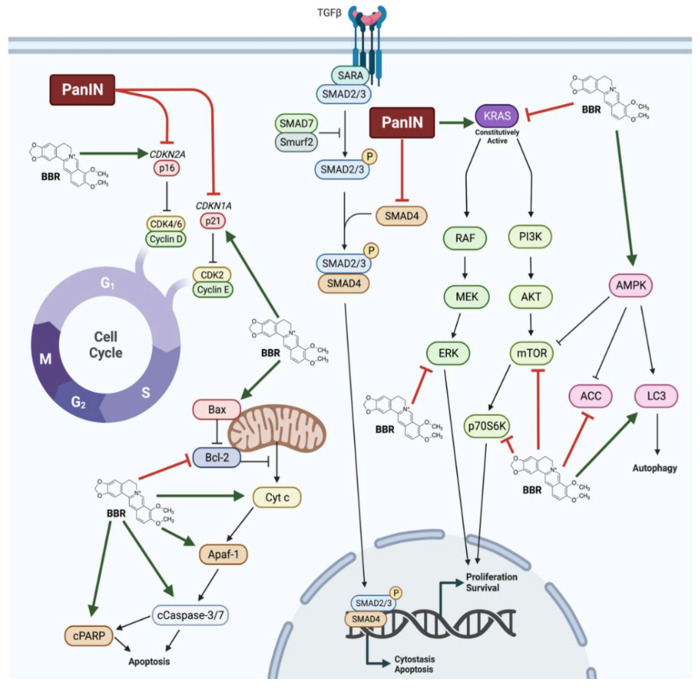
Summary of the effects of berberine in pancreatic cancer cells. Berberine (BBR) was shown to restore p16 and p21 function in pancreatic intraepithelial neoplasia (PanIN). BBR increased AMP-activated protein kinase (AMPK) and apoptosis signalling and decreased oncogenic Kirsten rat sarcoma virus (KRAS), extracellular signal-regulated kinase (ERK), mammalian target of rapamycin (mTOR), and ribosomal protein S6 kinase (p70S6K) signalling, culminating in decreased proliferation and survival, and increased apoptosis, cytostasis, and autophagy.

Several studies have investigated the effects of BBR in PanIN. In addition to inhibiting proliferation and survival of pancreatic cancer cells, BBR treatment was able to mitigate many of the cellular changes that are observed in PanIN. Notably, BBR restored p16 and p21 function, and inhibited oncogenic KRAS as well as downstream effectors of KRAS signalling including ERK, mTOR, and p70S6K (Figure 5). Additionally, BBR treatment activated AMPK signalling and initiated mitochondrial-mediated apoptosis. In animal models, BBR administered intraperitoneally or intragastrically inhibited tumour growth, decreased tumour metastasis, and prevented pancreatitis-induced malignant transformation. Taken together, these findings suggest significant effects of BBR against pancreatic cancer and point to the need of more animal studies and future clinical trials.

## 5. Conclusions

The effects of BBR against pancreatitis and pancreatic cancer have been summarized herein. The evidence is clear that BBR has beneficial effects in cellular and animal models of pancreatitis and pancreatic cancer. BBR administered intraperitoneally or intragastrically was able to prevent and reverse pancreatic tissue damage in animal models of pancreatitis. These effects coincided with a reduction in inflammatory markers including TNF-α, IL-6, and IL-1β, and a reduction in pro-inflammatory NFκB signalling. Since pancreatitis is a risk factor in malignant transformation leading to pancreatic cancer, the ability of BBR to prevent and reverse pancreatitis may have promising implications for cancer prevention.

## Figures and Tables

**Table 1:** Summary of the effects of berberine in animal models of pancreatitis.

Animal Model	Treatment	Effects	Signalling	Ref.
Taurocholate-induced SAP Sprague Dawley rats	Daily aqueous intragastric administration BBR 50 mg/kg for 5 days	↓ Intestinal barrier dysfunction	↓ phospho-MLC	[104]
		↓ Serum DAO activity		
		↓ Serum endotoxin levels		
		↓ Bacterial translocation		
Cerulein-induced AP and L-arginine-induced SAP C57BL/6 mice	Intraperitoneal injection BBR 1–20 mg/kg 1 h pre-treatment for 6 h	↓ Pancreatic edema	↓iNOS ↓NO ↓ TNF-α, IL-1β, and IL-6 ↓ phospho-JNK	[105]
		↓ Pancreatic inflammation		
		↓ Lung damage		
		↓Serum amylase and lipase		
		↓ Pancreatic myeloperoxidase		
		↓ pulmonary myeloperoxidase		
CDE diet-induced pancreatitis Female C57BL/6 mice	Intraperitoneal injection BBR 1, 5, 10 mg/kg/day For 3 days	↓ histological damage	↓ NFκB ↓ JNK ↓ p38	[106]
		↓ plasma amylase and lipase		
		↓ myeloperoxidase activity		
		↓ TNF-α, IL-1β, and IL-6		
		↓ mortality rate (60%)		
L-arginine-induced SAP Wistar rats	Intragastric administration BBR 100 mg/kg/day for 6 days	↑ Survival	-	[107]
		Protected from pancreatic encephalopathy		
		↓ pancreatic inflammation and necrosis		
		↓ amylase levels		
		Prevented blood-brain barrier permeability		
Cerulein-induced CP Male Swiss albino mice	Intraperitoneal injection BBR 3 or 10 mg/kg/day for 21 days	↑ pancreatic weight	↓ α-SMA ↓ collagen 1a and 3a ↓ fibronectin ↑ AMPK ↓ TGF-β1/Smad2/3 ↑ Smad7 ↑ E-cadherin ↓ Slug and Snail ↓ CD206	[100]
		↓ plasma amylase and lipase		
		↓ pancreatic MDA		
		↓ pancreatic nitrate		
		↑ GSH		
		↓ TNF-α, IL-6, IL-1β, and TGF-β1		
		↓ collagen deposition		
		↓ inflammatory cell infiltration		
		↓ acinar cell atrophy		
		↓ exocrine vacuolization		

Table legend: ↑ increases, ↓ reduces.

**Table 3:** Effects of BBR against pancreatic cancer in vivo.

Animal Model	Treatment	Effects	Ref.
Panc02 xenografted c57Bl/6 mice	Lovastatin:	↓ Tumour volume Synergistic effect	[109]
	Intraperitoneal injection 30 mg/kg/day		
	BBR: Oral administration 100 mg/kg/day for 14 days		
MiaPaCa-2 xenografted nude mice	Intraperitoneal injection	↓ Tumour weight ↓ Tumour volume	[110]
	5 mg/kg/day BBR		
AsPC-1 intravenous injection (tail vein) in BALB/c nude mice	Oral Gavage	↑ Survival ↓ Lung metastases ↓ Lung infiltration	[124]
	100–200 mg/kg/day BBR		
	Beginning 3 days prior to AsPC-1 injection		
Cerulein-induced CP/CP-induced neoplasia C57BL/6 mice	Intragastric Administration	↓ ADM ↓ PanIN ↓ Fibrosis ↓ CK19 ↑ Amylase	[127]
	BBR 200 mg/kg		
	Daily, 3 days/week		
	4–8 weeks		

Table legend: ↑ increases, ↓ reduces.
